# A virus‐induced gene‐silencing system for functional genetics in a betalainic species, *Amaranthus tricolor* (Amaranthaceae)

**DOI:** 10.1002/aps3.1221

**Published:** 2019-02-07

**Authors:** Dinesh Adhikary, Upama Khatri‐Chhetri, Fiona J. M. Tymm, Susan J. Murch, Michael K. Deyholos

**Affiliations:** ^1^ Department of Biology University of British Columbia Kelowna British Columbia Canada; ^2^ Agricultural, Food, and Nutritional Science Department University of Alberta Edmonton Canada; ^3^ Department of Chemistry University of British Columbia Kelowna British Columbia Canada

**Keywords:** Amaranthaceae, *Amaranthus tricolor*, *AtriCYP76AD1*, betalain, l‐DOPA, virus‐induced gene‐silencing (VIGS)

## Abstract

**Premise of the Study:**

Research in Amaranthaceae could be accelerated by developing methods for targeted gene silencing. Most amaranths, including *Amaranthus tricolor*, produce betalains. However, the physiological and ecological roles of these pigments are uncertain. We sought to establish a virus‐induced gene‐silencing (VIGS) method for amaranths, using silencing of betalain pigments as a proof‐of‐principle.

**Methods:**

We targeted *AtriCYP76AD1*, a putative cytochrome P450 component of the betalain biosynthetic pathway, using VIGS, and compared two different methods of introducing the VIGS construct into plants. We measured transcript abundance and concentrations of betalains and their l‐DOPA precursor in VIGS‐treated plants, and compared these to controls.

**Results:**

We observed that when *AtriCYP76AD1* was targeted by VIGS in normally red plants, *AtriCYP76AD1* and the related genes *AtriCYP76AD6* and *AtriCYP76AD5* had diminished transcript abundance. Furthermore, newly emergent petioles and leaves of VIGS‐treated plants appeared green, betacyanin accumulation was strongly reduced, and l‐DOPA accumulation was increased. No betaxanthin could be detected in this variety of *A. tricolor*, either before or after VIGS treatment.

**Discussion:**

These results help to establish the genetic basis of betalain synthesis in amaranths. Furthermore, this is the first report of VIGS in amaranths and demonstrates the potential of this technique for basic and applied research in these species.

Amaranth is an ancient crop in which scientific and commercial interest has recently been renewed. The *Amaranthus* L. genus contains about 70 species, three of which are cultivated for grain (*A. caudatus* L., *A. cruentus* L., and *A. hypochondriacus* L.), with some of the remainder being significant weeds (e.g., *A. palmeri* S. Watson, *A. retroflexus* L.) (Sauer, 1950; Costea and DeMason, 2001). Amaranth has advantages of being a protein‐rich C_4_ pseudo‐cereal that can be adapted to cultivation in a wide range of environments, with good tolerance of drought and salinity (Kong et al., 2009; Teng et al., 2015). It also has superior water use efficiency compared to many other C_3_ and C_4_ crops (Omamt et al., 2006; Huerta‐Ocampo et al., 2014). Amaranths naturally produce betalains, which may contribute to their agronomic and nutritional advantages. Some of these species have even been proposed as commercial sources of betalain pigment (Cai et al., 2005). Cultivated amaranths share many properties with quinoa (*Chenopodium quinoa* Willd.), which is likewise a cultivated member of the Amaranthaceae (Asao and Watanabe, 2010; Valcarcel‐Yamani and Da Silva Lannes, 2012).

Resources for molecular research in amaranths are gradually accumulating. Transcriptomes for some amaranths, as well as a whole genome sequence assembly for *A. hypochondriacus* (the major grain amaranth), have recently been made available (Délano‐Frier et al., 2011; Clouse et al., 2015). To make the most effective use of these sequence resources, reverse genetics protocols for amaranths are needed, as these allow hypotheses about the functions of specific genes to be tested. One of the most efficient methods for inducing loss‐of‐function in a targeted sequence is virus‐induced gene silencing (VIGS; Baulcombe, 1999). In VIGS, a recombinant viral genome that carries a small fragment of a targeted host gene is introduced into a host plant. As the recombinant genome spreads systemically, the host's post‐transcriptional gene‐silencing mechanism is guided in a sequence‐specific manner to repress gene expression (Baulcombe, 1999). Our objective is to establish an efficient protocol for VIGS in amaranths, using the visible pigments of the betalain biosynthetic pathway as a proof‐of‐principle.

Betalains are plant pigments unique to the order Caryophyllales (Strack et al., 2003; Brockington et al., 2011). Several chemical and biological studies have demonstrated their antioxidant activities (Cai et al., 2003; Borkowski et al., 2005). Earlier studies have also shown that the betalains from Amaranthaceae are stronger antioxidants than ascorbic acid, rutin, or catechins (Cai et al., 2003). Because these chemicals scavenge free radicals, they have potential to help prevent cancer and cardiovascular disease (Gengatharan et al., 2015). Betalains also have some uses in the pharmaceutical and food coloring industry (Azeredo, 2009). They are divided into two types: betacyanin (red, λ ≈ 536 nm) and betaxanthin (yellow, λ ≈ 480 nm) (Schwartz and Elbe, 1980; Cai et al., 1998; Kanner et al., 2001; Kugler et al., 2007; Goncalves et al., 2012). To date, a total of 78 different betalain pigments have been identified from 17 betalainic families (Khan and Giridhar, 2015). In beetroot, two of the pigments, betanin (λ ≈ 536 nm) and vulgaxanthin I (λ ≈ 480 nm) are the primary betacyanin and betaxanthin pigments, respectively (Nemzer et al., 2011; Goncalves et al., 2012). In the case of *Amaranthus*, information on chemical identity of betalains is still scant. Cai and colleagues (Cai et al., 1998) characterized betacyanin pigments from seven *Amaranthus* species, including *A. tricolor* L., and concluded that its betacyanins are mostly amaranthine (λ ≈ 535 nm). The chemical structure of amaranthine is similar to the structure of betanidin from red beets (Cai et al., 1998). Further study of betalains from amaranth is required to clarify the chemical and biological properties of these pigments.

Studies of betalain synthesis in beetroot (*Beta vulgaris* L.) have elucidated the role of two key enzymes, 4,5‐DOPA‐extradiol‐dioxygenase (DODA) and cytochrome P450 (CYP76AD1), in the synthesis of betaxanthin and betacyanin in that species (Christinet et al., 2004; Hatlestad et al., 2012). Silencing of the P450 cytochrome gene *CYP76AD1* caused the loss of red pigmentation and the appearance of yellow betaxanthin in beets (Hatlestad et al., 2012). Recently, transcriptome studies and post‐transcriptional gene silencing in *B. vulgaris* showed that two cytochrome P450 (CYP450) enzymes (CYP76AD5 and CYP76AD6) are involved in the conversion of l‐tyrosine to l‐DOPA (Polturak et al., 2016; Sunnadeniya et al., 2016). Studies of betalain biosynthesis in other betalainic species are needed to explore potential diversity in this pathway. In the present study, we have used VIGS to silence *AtriCYP76AD1* and investigated whether it is involved in the betalain biosynthetic pathway in *A. tricolor*.

## MATERIALS AND METHODS

### Plasmid construction

VIGS tobacco rattle virus (TRV) vectors (TRV1 and TRV2) (Ratcliff et al., 2001) were obtained from the Arabidopsis Biological Resource Center (ABRC; Columbus, Ohio, USA). Sequence of a predicted betalain biosynthetic gene of *A. cruentus* (*AcCYP76AD1* GenBank accession: KR376479.1) (Hatlestad et al., 2012) was obtained from the National Center for Biotechnology Information (NCBI) databases. Using BLAST (Altschul et al., 1990), the gene fragment was queried against an *A. tricolor* cv. Red Leaf (hereafter ‘Red Leaf’) transcriptome (Matasci et al., 2014). A 387‐bp coding region fragment was selected following the guidelines described by Senthil‐Kumar and Mysore (2014). The gene fragment of interest, *AtriCYP76AD1*, was ligated into the pTRV2 vector, and the VIGS construct was prepared (Appendix S1).

### Agroinoculum preparation and inoculation

**Figure 1:**
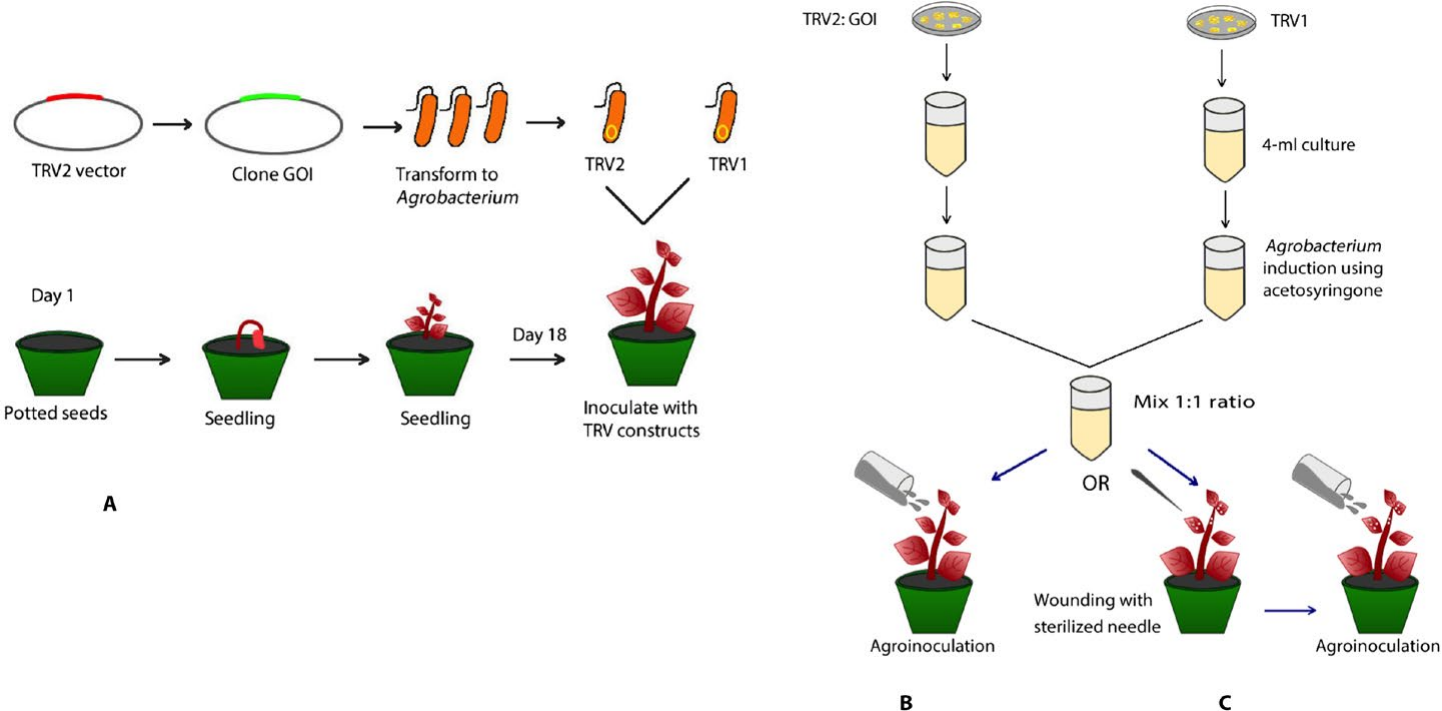
Tobacco rattle virus (TRV) inoculation methods in *Amaranthus tricolor* seedlings. (A) The gene of interest was synthesized and cloned into the TRV2 vector and transformed into *Agrobacterium* strain GV3101. (B) *Agrobacterium* cultures carrying TRV1‐ and TRV2‐*AtriCYP76AD1* were co‐inoculated in seedlings at 18 days after sowing, using the agroinoculation method, in which the inoculum was poured slowly from the apical region. (C) To improve the transformation efficiency, we introduced wounding with a sterilized needle, followed by the agroinoculation method.

Seeds of the intensely red‐pigmented variety *A. tricolor* ‘Red Leaf’ were obtained from Pias Blommor (Kalmar, Sweden). The species identification was confirmed through communication with David M. Brenner (Amaranth curator, U.S. Department of Agriculture–Agricultural Research Service Plant Introduction Research Unit [USDA‐ARS‐MWA‐PIRU], Iowa State University). A voucher specimen has been deposited at the University of British Columbia, Okanagan Campus (voucher DA 2018‐01). Seeds were germinated in plastic pots filled with potting mix and water in a growth chamber maintained at a temperature of 23°C with a 16/8 h light/dark photoperiod. For the VIGS assay, the bipartite TRV vector (pTRV) was used. The gene of interest, *AtriCYP76AD1*, was ligated into the pTRV2 vector. Both the pTRV1 and pTRV2 *AtriCYP76AD1* vectors were transformed into *Agrobacterium tumefaciens* strain GV3101 separately using a MicroPulser Electroporator (Bio‐Rad Laboratories, Hercules, California, USA). The agroinoculation procedure was carried out following the protocol outlined by Senthil‐Kumar and Mysore (2014) with a slight modification: 5 mL of culture was grown overnight at 28°C for both pTRV1 and pTRV2. In separate flasks, the fresh culture was inoculated into 300 mL of Luria–Bertani medium containing antibiotics. When the optical density of bacterial cells reached 0.6, cells were pelleted by centrifuging at 3000 rpm at room temperature (~22°C) for 25 min. The pellet was resuspended into an induction medium (pH 5.55). The culture was incubated for 3 h at room temperature in a shaker at a speed of 50 rpm. The induced cells were then harvested and resuspended in an inoculation medium (pH 5.55). The optical density of the inoculum was adjusted to 0.70 and inoculated into the 18‐d‐old seedlings, which had 3–4 leaves fully expanded and were 2.5–4 cm tall with stem diameter of 1.3 mm. Plants were grown under 350 μmol/m^2^ of light intensity in a growth chamber maintained at a temperature of 23°C with a 16/8 h photoperiod. A total of 300 seedlings were inoculated at four temporally independent replicates completed over a period of eight months. In each batch, plants were inoculated with an empty vector as a control, and a total of 210 plants were included in the experiment. We compared agroinoculation in wounded and non‐wounded plants. Prior to inoculation, seedlings to be wounded were randomly pierced with a sterilized needle on the leaf and stem. Each leaf was pierced in 5–7 spots on either side of the major vein and on the stem; 2–3 shallow scratches (0.5–1.0 cm in length, ~1.5 cm below the apical region) were created. Approximately 45–60 min after wounding, 20 mL of inoculum (optical density 0.70) was poured slowly from the apical region, making sure the stems and leaf portion were soaked (Fig. 1). Seedlings were kept covered overnight with a transparent plastic cover, and plants retained moisture on the leaf and stem surfaces. The next day, the plastic cover was removed. We also attempted vacuum infiltration, but no silencing was obtained by this method (data not shown).

### Reverse transcription real‐time PCR (RT‐qPCR) and confirmation of infection

Following the Minimum Information for Publication of Quantitative Real‐Time PCR Experiments (MIQE) guidelines, samples were prepared for RT‐qPCR and the experiment was performed (Bustin et al., 2009). Total RNA was extracted from VIGS phenotypic lines (TRV1/TRV2‐CYP76AD1), empty vector inoculated (TRV1/TRV2‐empty), and uninoculated groups. Fresh tissue samples were collected in liquid nitrogen and lysed into powder using TissueLyser II (QIAGEN, Valencia, California, USA), and total RNA was extracted using an RNeasy Plant Mini Kit (QIAGEN) following the manufacturer's protocol. On‐column DNAse digestion was performed to remove trace DNA using RNase‐free DNase (QIAGEN), and total RNA was quantified using an ND‐1000 Spectrophotometer (NanoDrop Technologies, Wilmington, Delaware, USA). RNA integrity was confirmed on a 1% agarose gel. First‐strand cDNA was synthesized using 1 μg of total RNA using iScript cDNA Synthesis Kit (Bio‐Rad Laboratories), and cDNA was stored at −20°C until use. To determine the transcript abundance of the target gene, RT‐qPCR was performed using SsoFast EvaGreen Supermix (Bio‐Rad Laboratories) following the manufacturer's protocol and *A. tricolor β‐actin* (*Atriβ‐Actin*) was used as the reference gene. Prior to running RT‐qPCR, primers for both the reference and target genes were optimized by running a gradient PCR, and the PCR amplicons were resolved in a 1.5% agarose gel. Using cDNA (1 : 5 dilution, 1.1 μL) as a template, melting curves were assessed at the best annealing temperature (*T*_a_ 57°C) for both genes, and quantification cycle (Cq) values were maintained at 21 cycles for the reference gene. A total of four independent experiments were carried out, and at least three biological replicates and three technical replicates were included for each treatment group in the RT‐qPCR. Using CFX Manager Software (Bio‐Rad Laboratories), normalized expression values were calculated and the fold change with respect to the uninoculated groups was calculated using Microsoft Excel (Microsoft Corporation, Redmond, Washington, USA). The significance of mean difference was analyzed by Student's *t*‐test in R version 3.3.3 (R Core Team, 2016), and the standard error was calculated.

To detect the presence of the TRV genome in the plant system, cDNA was used as a template in PCR with TRV1 and TRV2 gene‐specific primers. To confirm that the PCR amplicons for TRV were not derived from the *Agrobacterium* plasmids, primers outside the T‐DNA border were designed and PCR was performed on cDNA from the samples mentioned above.

To ensure that the right target had been captured in the RT‐qPCR, target‐specific primers were designed for *AtriCYP76AD1* (GenBank accession: KR376479.1) and *AtriCYP76AD6* (GenBank accession: KR376441.1) and PCR was performed. The amplified product was purified using GenElute PCR Clean‐up Kit (Sigma‐Aldrich Canada, Oakville, Ontario, Canada). The purified product was then sequenced by GenScript (GenScript, Piscataway, New Jersey, USA). Sequenced products were further aligned with the original target sequences (Appendix S2). All primer sequences used in this experiment are listed in Appendix 1.

### Betalain extraction and characterization using ultraviolet‐visible (UV‐Vis) spectrophotometry

Betalain pigment was extracted from VIGS‐treated plants that exhibited the loss of red pigment phenotype, uninoculated controls, an accession of *A. tricolor* with green leaves (accession: PI607446; obtained from USDA, Ames, Iowa, USA), and commercial red beetroot (obtained from a grocery store). Extraction followed the methodology outlined by Jain et al. (2015) with a slight modification: 0.1 g of fresh tissue was collected and kept frozen in liquid nitrogen, ground to a powder, resuspended in 5 mL of 100% methanol, and kept at 4°C for 2 h. The extracts were then centrifuged at 12,000 × *g* for 10 min. The supernatant was discarded, and the pellet was dissolved in 5 mL of reverse osmosis water (pH 5.0), as the betalain pigment is highly soluble in water. The absorption spectrum of the extracts was analyzed at the wavelength 280–730 nm at 23 ± 1°C on a UV‐Vis Spectrophotometer (UV‐6300PC; VWR International, Radnor, Pennsylvania, USA).

### l‐DOPA isolation and quantification

Samples were prepared following the protocol by Saremba et al. (2017) with minor modifications. In brief, 50 mg of leaf tissue was homogenized in a Kontes pellet pestle disposable tissue grinder (Thermo Fisher Scientific, Markhan, Ontario, Canada) in 200 μL of 80 : 20 (v/v) methanol (Fisher Optima LC/MS grade) : 0.1 N trichloroacetic acid (TCA). Samples were transferred into a centrifuge filter tube (0.2 μm, Ultrafree‐MC filtered centrifuge tubes; MilliporeSigma, Burlington, Massachusetts, USA) and centrifuged for 3 min at 16000 relative centrifugal force (RCF). The supernatant was transferred into a glass amber autosampler vial for chromatography using a validated ultra‐performance liquid chromatography–tandem mass spectrometry (UPLC–MS/MS) method. All sample preparation was performed in a dark room with a red light. l‐DOPA was separated in 10‐μL injections by reversed‐phase chromatography (30 × 3 mm, 2.6 μm C18 100 Å; Phenomenex, Torrance, California, USA) on a Waters ACQUITY I‐Class UPLC (Waters Inc., Mississauga, Ontario, Canada). The column temperature was set to 30°C. l‐DOPA was eluted with a gradient of 0.1% formic acid in e‐pure water (Eluent A; Sigma‐Aldrich Canada) and acetonitrile (Eluent B; Fisher Optima grade), with a flow rate of 0.3 mL/min and curve 6 mixing as follows: 90% A from 0.0–0.5 min, 40% A at 3.5 min, 5% A at 4.2 min, 5% A at 6.5 min, 90% A at 7.0 min.

A Xevo TQ‐S Quadrupole mass spectrometer (Waters Inc.) was used for detection and quantification, with electrospray ionization in ES+ mode. The capillary voltage was 3.50 kV, and the cone voltage was 55.00 V with an offset of 30.0 V. The source temperature was set to 150°C with a desolvation temperature of 550°C. Nitrogen was used as the cone gas and desolvation gas with a flow rate of 150 L/h and 800 L/h, respectively. Argon gas was used for the collision cell with a flow rate of 0.15 mL/min. The nebulizer gas flow was set to 7.00 bar. Conditions for multiple reaction monitoring of ion transitions were optimized with MassLynx version 4.1 (Waters Inc.). Data were acquired by MassLynx version 4.1, processed using TargetLynx version 4.1 (Waters Inc.), and exported to Excel. Details of l‐DOPA isolation and quantification are given in Appendix S3.

Two independent experiments were conducted with at least three biological replicates for each treatment group. l‐DOPA was quantified from each biological replicate and averaged. The significance of mean difference was analyzed by Student's *t*‐test in R, and the standard error was calculated.

### Phylogenetic analysis

The target sequence, *AtriCYP76AD1* (contig17897), and potential homologs (contig14699, contig17428, contig83066, contig15583, and contig74588) were obtained from the 1KP project (Matasci et al., 2014). Using a BLAST search, similar sequences were searched from NCBI databases and best‐hit sequences of betalainic plants were selected. The betalainic species included in the phylogeny are as follows: KR376385.1 *Froelichia latifolia*; KR376382.1 *Alternanthera caracasana*; KR376378.1 *Alternanthera ficoidea*; KT962274.1 *Beta vulgaris*; KR376381.1 *Beta vulgaris* subsp. *maritima*; HQ656026.1 *Mirabilis jalapa*; HQ656025.1 *Amaranthus cruentus* cv. Kerala; HQ656023.1 *Beta vulgaris* cv. W357B; KC857455.1 *Celosia cristata*, and KM592961.1 *Beta vulgaris*. Using MUSCLE 3.8 (Edgar et al., 2004), multiple sequence alignments of *AtriCYP76AD6* and *AtriCYP76AD1*‐like nucleotide sequences were created, and MEGA7.0 (Kumar et al., 2018) was used to process the maximum likelihood method and obtain a phylogenetic tree.

## RESULTS

### Tobacco rattle virus can induce silencing of *Amaranthus tricolor* genes

Successful VIGS requires systemic propagation of TRV components throughout the plant. To test whether TRV could be propagated in *A. tricolor*, we transformed TRV‐RNA1 (pTRV1) and TRV‐RNA2 (pTRV2) T‐DNA vectors separately into *Agrobacterium tumefaciens* (Ratcliff et al., 2001). These vectors together provide the movement and replication proteins and other viral factors required for VIGS. We then inoculated a mixture of cultures of the pTRV1 and pTRV2 transformants into *A. tricolor* plants at 18 days after germination (Fig. 1). Twenty‐one days post‐infection, leaves were collected from the new growth at the apex of the plant, i.e., from tissues that appeared subsequent to the inoculation. Using PCR with primers specific to TRV sequences, we were able to detect components of the TRV genome within the cDNA extracted from these leaves, whereas no amplification was obtained from plants that had not been inoculated (Appendix S4: B, C). To further verify that the cDNA amplification was not due to plasmid contamination, primers specific to the plasmid vector, but outside of the T‐DNA borders, were found to be unable to amplify cDNA (Appendix S4: D, E). These results showed that TRV successfully replicated and spread systemically throughout the inoculated plants.

**Figure 2:**
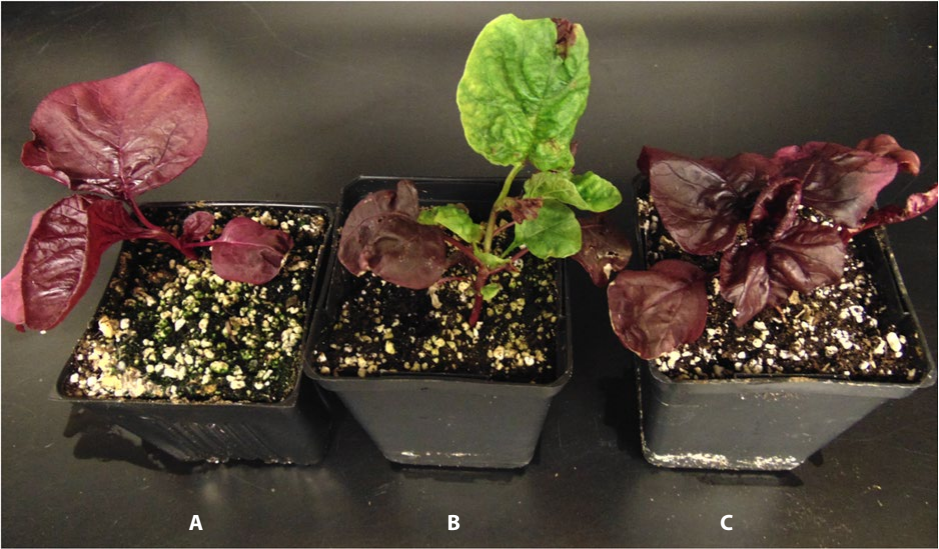
Virus‐induced gene‐silencing (VIGS) phenotypes in *Amaranthus tricolor* inoculated with TRV1‐ and TRV2‐*AtriCYP76AD1*. Whole plants representative of uninoculated (A), TRV1/TRV2‐*AtriCYP76AD1*–inoculated (B), and TRV1/TRV2 empty vector–inoculated (C) *A. tricolor* ‘Red Leaf’ at 21 days post‐inoculation.

To test whether TRV‐based VIGS could be used to silence endogenous genes in amaranths, we inserted a 387‐bp fragment of the *A. tricolor* cytochrome P450 gene *AtriCYP76AD1* into vector pTRV2 (Appendix S5). *AtriCYP76AD1* is the presumptive homolog of *Beta vulgaris CYP76AD1*, which is required for synthesis of betacyanin in beets. We inoculated a mixture of *A. tumefaciens* cultures containing pTRV1 and pTRV2‐*AtriCYP76AD1* into *A. tricolor* ‘Red Leaf’ seedlings. Two methods were used to inoculate the plants (Fig. 1B, C): (1) agroinoculation and (2) wounding followed by agroinoculation. Wounding was motivated in part by a report showing the increased efficiency of silencing when wounding was introduced into the tomato plants (Liu et al., 2002). In both cases, approximately 21 days post‐inoculation, a strong reduction in red pigments was observed in new growth of some inoculated plants, and the emergence of VIGS phenotypic leaves continued for at least 48 days post‐inoculation. The reduction in red pigmentation persisted until the senescent leaves fell from the plants. No silencing was observed within the inflorescences of the plants, and progeny appeared to have parental, non‐silenced pigmentation in their seed surfaces. The agroinoculation method produced silencing with 6% efficiency, compared to wounding followed by agroinoculation, which resulted in silencing on average in 45% of plants in three independent experiments (Appendix 2). Silencing resulted in green leaves and petioles (Fig. 2).

**Figure 3:**
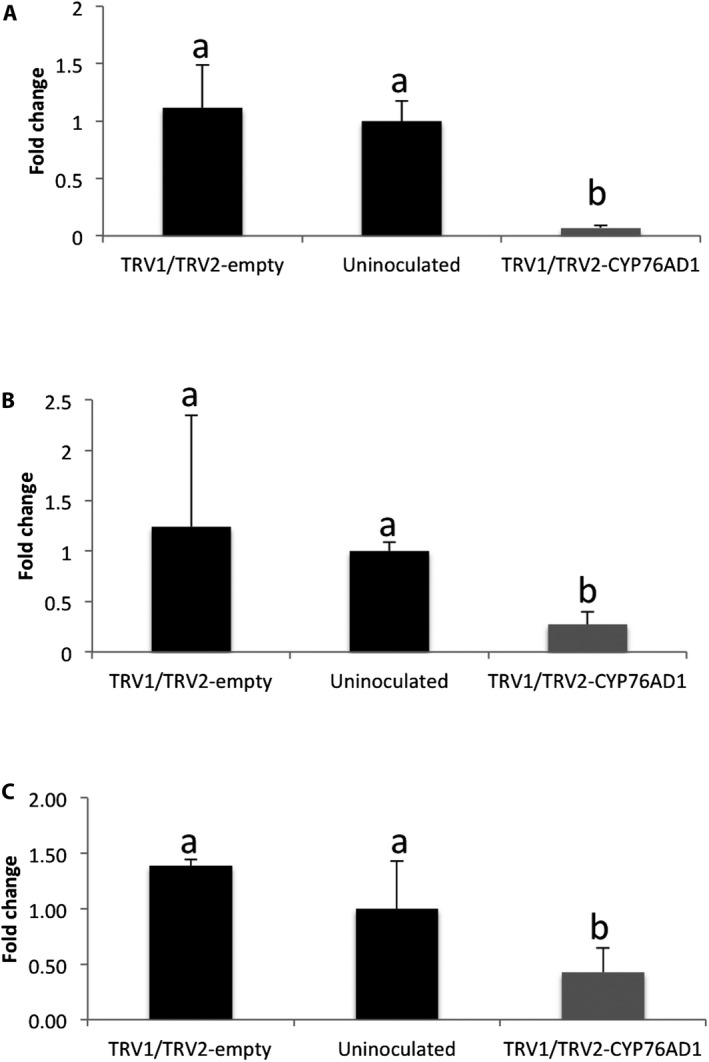
Transcript expression of *AtriCYP76AD1* (A), *AtriCYP76AD5* (B), and *AtriCYP76AD6* (C) measured by RT‐qPCR. Plants were inoculated either with an empty vector (TRV1/TRV2‐empty), a VIGS construct (TRV1/TRV2‐*AtriCYP76AD1*), or were uninoculated. From the TRV1/TRV2‐*AtriCYP76AD1*–inoculated group, only plants that showed a silencing phenotype (i.e., visible loss of red pigment) were included in this analysis. Values are means ±SE (*n* = 3). Fold change in all groups were averaged and plotted in the bar graphs. Lowercase letters a and b show the statistical significance (*P* < 0.05) in the mean differences between the treatments.

To verify that VIGS resulted in suppression of the targeted transcript, we used quantitative RT‐qPCR to measure transcript abundance in green leaves of pTRV2‐*AtriCYP76AD1*–inoculated plants. The target gene showed significantly reduced transcript abundance in the VIGS phenotypic plant (TRV1/TRV2‐CYP76AD1) compared to the uninoculated and empty vector–inoculated (TRV1/TRV2‐empty) plants (Fig. 3A). The difference between the empty vector–inoculated and the uninoculated group was not statistically significant (Fig. 3A). These results showed that the VIGS treatment was successful in suppressing the expression of the targeted transcripts in VIGS phenotypic groups.

Analysis of *BvCYP76AD1* and related genes within the Caryophyllales suggested that multiple gene duplication events have produced three clades within the *CYP76AD1* lineage (*CYP76AD1‐α*,* CYP76AD1‐β*, and *CYP76AD1‐γ*) (Brockington et al., 2015). In the current study, the *AtriCYP76AD1* target gene clusters in the same clade as *BvCYP76AD1* (Appendix S6). Pairwise alignment of these two genes showed 83% identity in amino acid sequence (Appendix S7). *AtriCYP76AD1* and *BvCYP76AD1* are reciprocal best BLAST matches, and from the *A. tricolor* transcriptome assembly we selected the next best BLAST hits to *BvCYP76AD1* (contig74588, contig14699, contig17428, contig15583, contig83066) and included these in the dendrogram. These contigs were clustered in different clades (Appendix S6). We selected two contigs (contig74588 and contig14699) from different clades to test for the off‐target effects and measured their transcript abundance in the *AtriCYP76AD1* VIGS plants. Contig74588 and contig14699 are potential homologs of *BvCYP76AD5* and *BvCYP76AD6*, respectively. Pairwise alignments of *AtriCYP76AD1* to contig74588 and contig14699 revealed 77% and 67% identity, respectively, in protein sequence (Appendix S8). We observed that the transcript abundance of contig74588 (*AtriCYP76AD5*) and contig14699 (*AtriCYP76AD6*) was also significantly decreased as compared to the uninoculated and empty vector–inoculated groups (Fig. 3), indicating that some off‐target silencing had occurred.

### Betalain quantification from normal and transgenic plants

**Figure 4:**
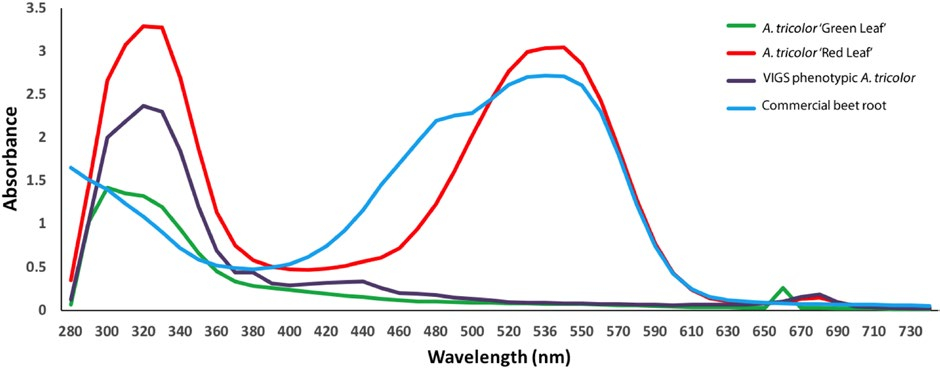
Absorbance spectrum in the UV‐visible range of amaranth extracts taken from leaves of pigmented wild‐type and unpigmented VIGS *Amaranthus tricolor* plants. Using a spectrophotometer, absorbance was measured in aqueous extracts from 280–730 nm at 1‐nm resolution. Untreated *A. tricolor* ‘Red Leaf’ showed an absorbance maximum in the red range at 536 nm, but no detectable peak in the yellow range at 470–480 nm. However, extract from commercial beetroot had detectable peaks at both 536 nm and 480 nm. Data shown are representatives of two replicates. Green leaves from VIGS‐treated *A. tricolor* ‘Red Leaf’, or from a green accession of *A. tricolor* did not show absorbance at either 536 nm or 480 nm.

We used UV‐Vis spectrophotometry of leaf extracts to further characterize the impact of *AtriCYP76AD1* VIGS on the betalain pathway in *A. tricolor*. We observed peak absorbance in *A. tricolor* ‘Red Leaf’ at 536 nm (Fig. 4). Interestingly, there was no evidence of a betaxanthin‐associated peak in *A. tricolor* ‘Red Leaf’, even though we could detect absorption near 480 nm in the spectra we obtained from red roots or red petioles of *Beta vulgaris* (Fig. 4). The green, *AtriCYP76AD1*‐silenced *A. tricolor* leaves showed no detectable absorption peaks at either 480 nm or 536 nm, indicating a complete absence of detectable betacyanins and betaxanthins. In this respect, the spectrum of *AtriCYP76AD1* VIGS *A. tricolor* leaves was indistinguishable from extracts of the green, ‘Green Leaf’ cultivar of *A. tricolor*. The lack of detectable betacyanins in the VIGS phenotypic tissue is consistent with a central role for *AtriCYP76AD1* and *AtriCYP76AD6* in betalain synthesis (Figs. 2 and 4).

### l‐DOPA accumulation in the transgenic plants

**Figure 5:**
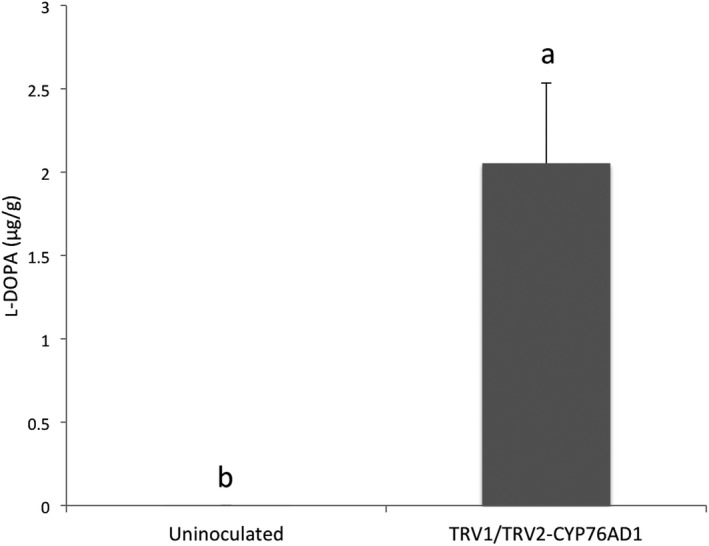
The concentration of l‐DOPA measured in leaves of uninoculated *Amaranthus tricolor* ‘Red Leaf’ and in the green leaves that appeared on *A. tricolor* ‘Red Leaf’ after inoculation with TRV1/TRV2‐*AtriCYP76AD1* using ultra‐performance liquid chromatography. Values are means ±SE (*n* = 5). Lowercase letters a and b show the statistical significance (*P* < 0.05) in the mean differences between the treatments.

Because the silencing of *AtriCYP76AD1* in *A. tricolor* produced an accumulation of pigments that differed from previous reports of silencing of *CYP76AD1* in other species, we sought to quantify l‐DOPA, which is the presumed substrate of *AtriCYP76AD1* (Hatlestad et al., 2012). Using UPLC, we measured 2.05 μg/g of l‐DOPA in the *AtriCYP76AD1* VIGS plants, which is a statistically significant increase compared to the 2.75 × 10^−5^ μg/g l‐DOPA measured in non‐silenced *A. tricolor* ‘Red Leaf” (Fig. 5). At different times, the experiment was repeated twice, independently, using experimental triplicates, and the same pattern was observed. The accumulation of l‐DOPA in VIGS plants supports the assumption that AtriCYP76AD1 uses l‐DOPA as a substrate in *A. tricolor* ‘Red Leaf’, consistent with previous reports that CYP76AD1 and CYP76AD6 can metabolize l‐DOPA in beetroot (Hatlestad et al., 2012; Polturak et al., 2016).

## DISCUSSION

Our major objective was to establish the VIGS method in amaranth and demonstrate the function of *AtriCYP76AD1* in the betalain biosynthesis pathway in *A. tricolor* (Appendix S9). We found that wounding, followed by agroinoculation of TRV‐based constructs, resulted in an average efficiency of silencing of 45% in *A. tricolor*. Wounded plants had a higher efficiency of silencing than non‐wounded plants. This approached the efficiency reported for suppression of the phytoene desaturase gene (*PDS*) in tomato plants (Liu et al., 2002) and confirms the utility of this silencing technology for reverse genetics in amaranths. The VIGS effect persisted in the tissues in which it was observed, but was not heritable.

The visible reduction in red pigmentation following inoculation with *AtriCYP76AD1* and the spectrophotometric analysis of leaf extracts confirmed that *AtriCYP76AD1* is necessary for production of red betalain pigments in *A. tricolor*. *AtriCYP76AD1* is the best reciprocal BLASTN match for *B. vulgaris CYP76AD1*, which was shown to be necessary for production of betacyanin in beet petioles and roots, and was also sufficient for production of betacyanin from l‐DOPA when expressed heterologously in yeast (Polturak et al., 2016). However, silencing of *CYP76AD1* in *B. vulgaris* resulted in a change from red to visibly yellow tissues (Hatlestad et al., 2012), which is different than the change from red to visibly green tissues we observed in *A. tricolor* ‘Red Leaf’ VIGS plants (Fig. 2). In fact, in *B. vulgaris*, green tissues could be produced only by silencing a different enzyme, DODA, or in a separate experiment by silencing both *CYP76AD1* and *CYP76AD6* (Polturak et al., 2016). The green appearance of our *AtriCYP76AD1* VIGS plants could be explained by off‐target silencing of *AtriCYP76AD6*, and indeed we observed a significant decrease in *AtriCYP76AD5* and *AtriCYP76AD6* transcripts in our RT‐qPCR assays (Figs. 3B, C). However, this model does not explain the very strong increase in l‐DOPA accumulation we observed in our *AtriCYP76AD1* VIGS plants, as *CYP76AD1* and *CYP76AD6* are the two genes that have been demonstrated to produce l‐DOPA from tyrosine in *B. vulgaris* (Polturak et al., 2016). We therefore hypothesize that: (1) residual activity of CYP76AD6 or other enzymes in VIGS plants is able to catalyze production of l‐DOPA, and (2) the ‘Red Leaf’ cultivar of *A. tricolor* that we used is these experiments is unable to convert l‐DOPA into betaxanthins; this may allow the accumulation of l‐DOPA when betacyanin production is blocked in *AtriCYP76AD1* VIGS plants. These hypotheses are supported by the accumulation of l‐DOPA in *AtriCYP76AD1* VIGS plants and by the lack of detectable absorbance near 480 nm in the absorbance spectrum of untreated *A. tricolor* ‘Red Leaf’ extracts (Figs. 4 and 5). Although a 480‐nm absorbance peak could conceivably be hidden by the shoulder of the large peak at 536 nm in *A. tricolor*, an influence of a 480‐nm peak is clearly visible in *B. vulgaris* extracts, indicating that betacyanins and betaxanthins can be easily detected simultaneously in at least some visibly red species.

Several organisms have been evaluated for their potential as commercial sources of l‐DOPA (Rani et al., 2007; Patil et al., 2013), including suspension cultures of plant and fungal cells (Ali et al., 2002; Sikander, 2006; Rani et al., 2007) and seeds of legumes including *Mucuna pruriens* (L.) DC. (velvet bean) (Egounlety, 2003) and *Vicia faba* L. (faba bean). Yields of up to 22.4 mg/g l‐DOPA have been reported in leaves of faba bean (Etemadi et al., 2018) and up to 63.6 mg/g by weight in seeds of velvet bean (Egounlety, 2003). Compared to these legumes, we observed substantially less (2.05 μg/g) l‐DOPA in *AtriCYP76AD1* VIGS plants (Fig. 5), meaning that although manipulation of betalain biosynthesis in *A. tricolor* substantially increased l‐DOPA concentration, this is unlikely to be a competitive strategy for industrial‐scale production of this metabolite. The increased accumulation of l‐DOPA that we observed when *AtriCYP76AD1* was silenced in *A. tricolor* ‘Red Leaf’ could be interpreted in the following ways: (1) the reaction catalyzed by *AtriCYP76AD1* is the main (or possibly only) sink for l‐DOPA, which calls into question the role for DODA in the synthesis of betalains in *A. tricolor* ‘Red Leaf’; or (2) DODA activity, and not the availability of l‐DOPA, is the limiting step in the synthesis of betalamic acid. However, distinguishing between these possibilities will require further research.

We have established that TRV‐based VIGS in *A. tricolor* can be used for efficient reverse genetics experiments in this species and presumably its close relatives. It is also noteworthy that the leaves with reduced pigmentation continued to show this same phenotype through senescence; therefore, it is possible that VIGS *A. tricolor* plants could be used for other in vivo experiments to establish physiological and ecological roles for betalains. This work also confirms the central role of *AtriCYP76AD1* in betacyanin synthesis and raises questions about betaxanthin synthesis in *A. tricolor* ‘Red Leaf’.

## AUTHOR CONTRIBUTIONS

D.A. and M.K.D. conceived and designed the experiments. D.A. and F.J.M.T. carried out the experiments. D.A., M.K.D., and U.K.C. performed the data analysis. D.A. wrote the manuscript; U.K.C., F.J.M.T., S.J.M., and M.K.D. provided editorial advice. All authors read and approved the final manuscript.

## Supporting information


**APPENDIX S1.** Plasmids used in virus‐induced gene‐silencing (VIGS) experiments in *Amaranthus tricolor*. pTRV1 and pTRV2 are as described in Senthil‐Kumar and Mysore (2014).

**APPENDIX S2.** Confirmation of RT‐qPCR target by sequencing. (A) Alignment of *AtriCYP76AD1* RT‐qPCR product with the original reference. (B) Alignment of *AtriCYP76AD6* RT‐qPCR product with the original reference.

**APPENDIX S3.** Detailed description of l‐DOPA isolation and quantification method.

**APPENDIX S4.** PCR to confirm the silencing of the target in *Amaranthus tricolor*. (A) Actin was used as an internal control to check the quality of cDNA synthesized from plant tissues. In all panels, a 100‐bp DNA ladder was used as the marker (M). Lanes 1–3 indicate the empty vector group replicates, TRV1/TRV2‐empty (labeled as EL); lanes 4–6 indicate the uninoculated group replicates (labeled as RL); lanes 7–9 indicate the replicate of the VIGS phenotypic group, TRV1/TRV2‐*AtriCYP76AD1* (labeled as VL). Lane 10 is a no template control (labeled as –ve). (B) TRV2‐specific primers were used to confirm the presence of viral genome in the EL, RL, and VL groups. Lane 10 is the negative control in the panel. (C) TRV1‐specific primers were used to confirm the presence of viral genome in the VIGS plants. Lane 10 shows the amplicons from the positive control, TRV1 plasmid. Lane 11 indicates the no template control. Two TRV‐specific targets were selected outside the T‐DNA border to test the plasmid contamination on cDNA. (D) NPTII gene primers or kanamycin‐resistance gene (represented as KanR in Appendix S1) in lanes 10 and 11 indicate the TRV2 plasmid as the positive control and the no template control, respectively. (E) DNA fragment outside the T‐DNA border (TRV2‐offBorder) in lanes 10 and 11 indicate plasmid DNA from TRV1 and TRV2 plasmids indicating positive control, and lane 12 shows the no template control.

**APPENDIX S5.** The DNA sequence of the fragment inserted into pTRV2 to target *CYP76AD1* for virus‐induced gene silencing (VIGS).

**APPENDIX S6.** Multiple sequence alignment of *CYP76AD1*‐like gene sequences. The dendrogram is obtained from a maximum likelihood method using nucleotide sequences of *Amaranthus tricolor* transcript sequences (contig74588, contig83066, contig14699, contig17428, contig17897, and contig15583). The dark arrows point at the gene sequences that were used in RT‐qPCR for their transcript quantification. * indicates the VIGS target, *AtriCYP76AD1*. Contig74588 and contig14699 are potential homologs of *BvCYP76AD5* and *BvCYP76AD6*, respectively. The betalainic species included in the phylogeny are as follows: KR376385.1 *Froelichia latifolia*; KR376382.1 *Alternanthera caracasana*; KR376378.1 *Alternanthera ficoidea*; KT962274.1 *Beta vulgaris*; KR376381.1 *Beta vulgaris* subsp. *maritima*; HQ656026.1 *Mirabilis jalapa*; HQ656025.1 *Amaranthus cruentus* cv. Kerala; HQ656023.1 *Beta vulgaris* cv. W357B; KC857455.1 *Celosia cristata*; and KM592961.1 *Beta vulgaris*.

**APPENDIX S7.** Sequence alignment of *AtriCYP76AD1* and *BvCYP76AD1*.

**APPENDIX S8.** Protein sequence alignment. (A) *AtriCYP76AD1* and *AtriCYP76AD6*. (B) *AtriCYP76AD1* and *AtriCYP76AD5*.

**APPENDIX S9.** Proposed betalain biosynthetic pathway. Steps 1, 2, and 3 are enzyme mediated; steps 4 and 5 are proposed to be spontaneous. Pathway is redrawn from Hatlestad et al. (2012).

## APPENDIX 1 Primer sequences used in this study.

**Table:**

Primer name	Forward sequence	Reverse sequence
*Atriβ‐Actin* *	CTGCCATTGAGAAGAACTAC	AGCTTCCATACCGATCAA
*AtriDODA* *	CACACTTGGATGGCATGTA	GGAGCGACACCATCATAATAA
*AtriCYP76AD1* *	CCAAGGTGCAACAACTTTATC	GAGATTTATGGCAAGCTAATTCC
*AtriCYP76AD6* *	AGGGAGAAGCTCTTGATATT	CCCGAATCCCTAGAACTATAA
*TRV1* **	CGGGCAAATGATGCAAAGAG	ACCGTCAACAGAAGGTAATGG
*NPTII* **	GCCAACGCTATGTCCTGATA	GATCTCCTGTCATCTCACCTTG
*TRV2‐offBorder* **	CTGGGAGACAGCAACATCTTT	CGGCTACACTAGAAGGACAGTA
TRV2_coat protein**	ACTCACGGGCTAACAGTGCT	GACGTATCGGACCTCCACTC

*Primers used for RT‐qPCR.

**Primers used for regular PCR.

## APPENDIX 2 Efficiency of virus‐induced gene‐silencing (VIGS) inoculation methods.

**Table:**

Method	Days post‐infection	Phenotype observed	Total individuals treated	% Efficiency
Agrodrench	21	Yes	75	6%
Wounding and agrodrenching	21	Yes	125	73%
Wounding and agrodrenching	23	Yes	75	33%
Wounding and agrodrenching	20	Yes	75	29%
