# Supplementary material for: A virus‐induced gene‐silencing system for functional genetics in a betalainic species, Amaranthus tricolor (Amaranthaceae)
Source: Appl Plant Sci. 2019 Feb 7;7(2):e01221. doi: 10.1002/aps3.1221 (PMC6384298; doi:10.1002/aps3.1221)

Adhikary et al.—Applications in Plant Sciences 2019 7(2)—Data Supplement S1
DOI

APPENDIX S1. Plasmids used in virus-induced gene silencing (VIGS) experiments in *Amaranthus tricolor*. pTRV2 is as described in Senthil-Kumar and Mysore (2014).

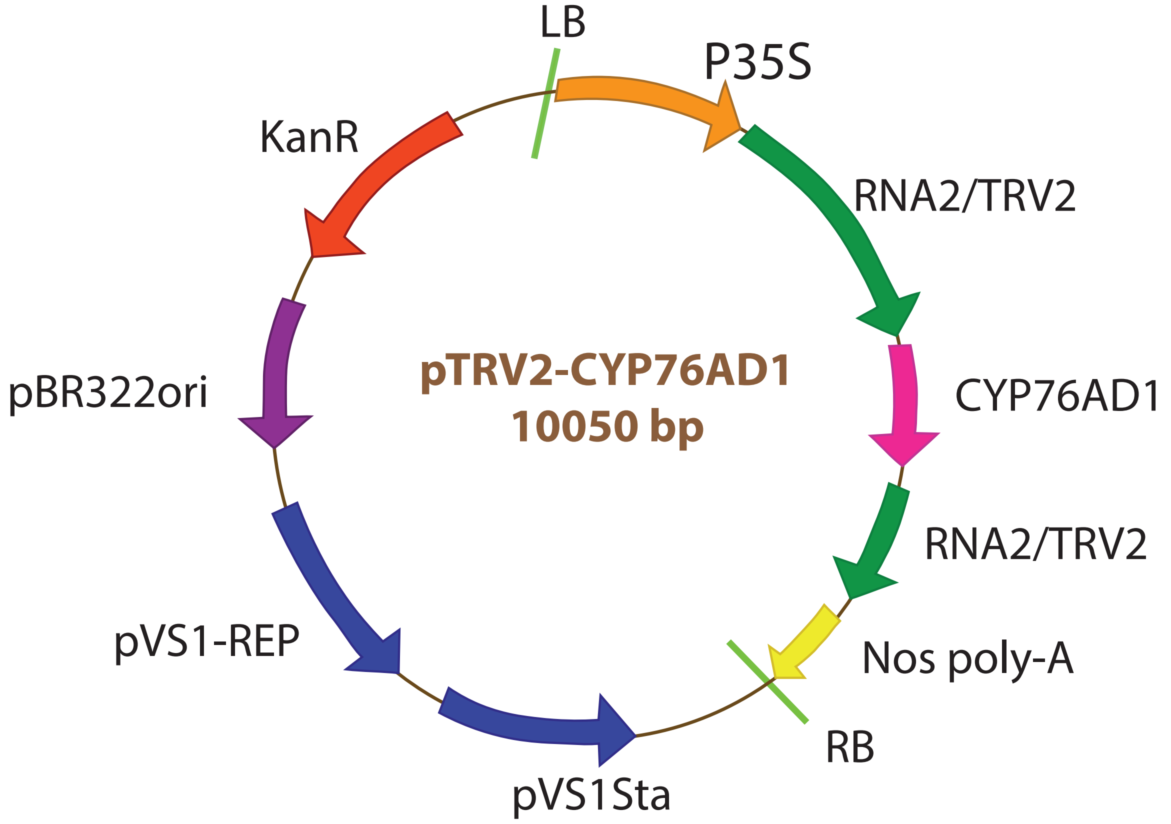

Supplement: Supplementary file 1 — APPENDIX S1. Plasmids used in virus‐induced gene‐silencing (VIGS) experiments in Amaranthus tricolor. pTRV1 and pTRV2 are as described in Senthil‐Kumar and Mysore (2014). [file APS3-7-e01221-s001.pdf]
