# Supplementary material for: A virus‐induced gene‐silencing system for functional genetics in a betalainic species, Amaranthus tricolor (Amaranthaceae)
Source: Appl Plant Sci. 2019 Feb 7;7(2):e01221. doi: 10.1002/aps3.1221 (PMC6384298; doi:10.1002/aps3.1221)
Supplement: Supplementary file 3 — APPENDIX S3. Detailed description of l‐DOPA isolation and quantification method. [file APS3-7-e01221-s003.pdf]

DOI

APPENDIX S3. Detailed description of L-DOPA isolation and quantification method.

Preparation of samples

Previously described and validated analytical methods were adapted for isolation and quantification of L-DOPA (Saremba et al., 2017). In brief, a 50-mg leaf tissue sample was homogenized (Kontes Pellet Pestle disposable tissue grinder; Thermo Fisher Scientific, Waltham, Massachusetts, USA) in a dark room with a red light in 200 µL of 80 : 20 (v/v) methanol : 0.1 N trichloroacetic acid (TCA). Samples were transferred into a centrifuge filter tube (0.2 µm, Ultrafree-MC filtered centrifuge tubes; MilliporeSigma, Burlington, Massachusetts, USA) and centrifuged for 3 min at 16,000 *g*. The supernatant was transferred into a glass insert in a glass amber autosampler vial for chromatography. L-DOPA was quantified in a 10-µL injection of each sample.

Preparation of standards

The L-DOPA standard (Sigma-Aldrich Canada, Oakville, Ontario, Canada) was prepared in the previously described dark conditions. In brief, a 2.5-µmol/mL stock solution (L0) of L-DOPA was diluted in series to create a 6-point calibration curve with a range of 19256.63–616212.19 pg on column after a 10-µL injection. An initial 1 : 4 dilution was made in Eppendorf tubes (Eppendorf, Hamburg, Germany) by taking 25 µL of L0 and diluted in 75 µL of 80% methanol (MeOH) in 0.1 N TCA, which resulted in L2. L2 was then vortexed and 50 µL was taken and mixed with 50 µL of the 80 : 20 (v/v) MeOH : 0.1 N TCA solution, which resulted in L3. A dilution series was produced following the same 1/2 dilutions until the desired concentration of L10 was obtained. The remaining 75 µL of each standard was transferred to a conical glass insert in an amber autosampler vial. The precision of L-DOPA retention on column was 0.28% relative standard deviation.

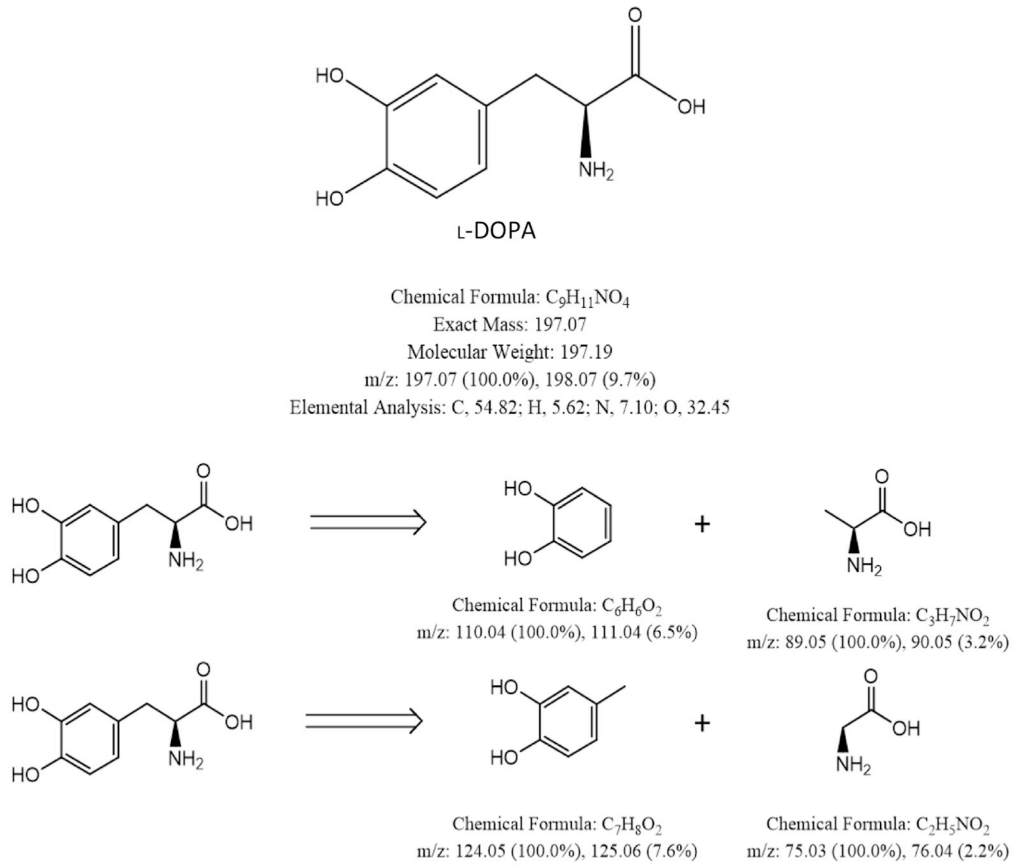

FIGURE S3-1. Predicted fragmentation of L-DOPA by ChemDraw.

Chromatographic separation of analytes

All samples were separated on a reverse phase column (30 × 3 mm, 2.6 μm C18 100 Å; Phenomenex, Torrance, California, USA) using Waters Acquity I-Class Ultra-performance liquid chromatography (UPLC; Waters Inc., Mississauga, Ontario, Canada). The column temperature was set to 30°C. Mobile phase A was 0.1% formic acid (Sigma-Aldrich Canada) in e-pure water. Mobile phase B was 100% acetonitrile. With a flow rate of 0.3 mL/min and an eluent mixing curve of 6, the gradient used was: 0:00 min, 10% B; 0.50 min, 10% B; 3.50 min, 60% B; 4.20 min, 95% B; 5.20 min, 95% B; 7:00 min, 10% B. The entire flow rate was sent to the mass spectrometer for the duration of the run. The method was selective for L-DOPA, and baseline separation was achieved. A 90% acetonitrile in e-pure water solution was used for all of the wash solvents for the instrument for this method.

Mass spectrometry detection

A Xevo TQ-S Quadrupole mass spectrometer (Waters Inc.) was used for detection and quantification, with electrospray ionization (ESI) in ES+ mode. The capillary voltage was 3.50 kV and the cone voltage was 55.00 V with an offset of 30.0 V. The source temperature was set to 150°C with a desolvation temperature of 550°C. Nitrogen was used as the cone gas and desolvation gas with a flow rate of 150 L/h and 800 L/h, respectively; and argon gas was used for the collision cell with a flow rate of 0.15 mL/min. The nebulizer gas flow was set to 7.00 bar. Conditions for multiple reactions monitoring of ion transitions were optimized with MassLynx version 4.1 (Waters Inc.).

Determination of fragmentation pattern

Fragmentation transitions predicted by ChemDraw (PerkinElmer, Waltham, Massachusetts, USA) included 197.19 m/z to 124.05 m/z, 197.19 m/z to 110.04 m/z, 197.19 m/z to 89.05 m/z, and 197.19 m/z to 75.03 m/z (Fig. S3-1, S3-2). Detection conditions were optimized for the potential fragments.

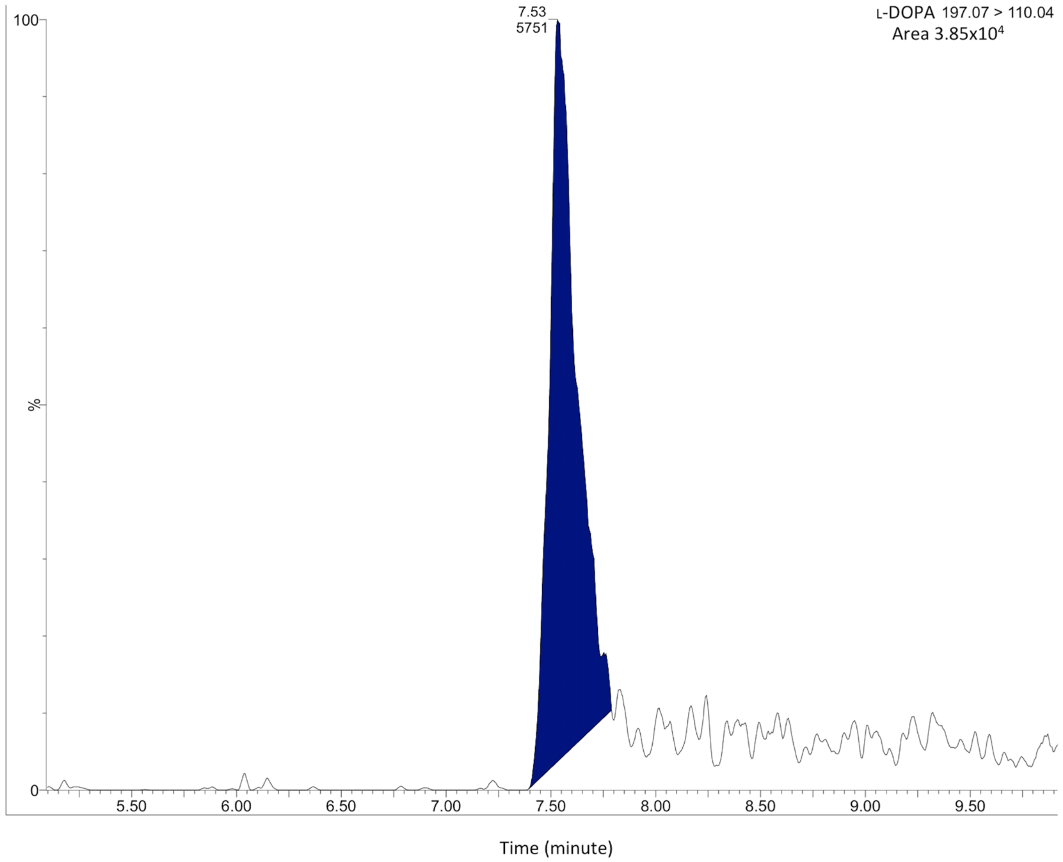

FIGURE S3-2. Separation and quantification of the L-DOPA peak by the 197.07 m/z to 110.04 m/z transition.

A direct infusion of standards was used to determine response and fragmentation parameters. The data were acquired over 10 s during the infusion to collect the peak area of the total ion count to determine relative responses. For the fragmentation transitions of 197.19 m/z to 124.05 m/z, the cone voltage was first optimized increasing in increments of 5 V from 10 V up to 120 V, while the collision was held at 1 V. The collision was then tested in intervals of 5 from 1 to 70 V, which shortened the range, allowing the collision to run from 1–10, optimized at 2 V. The same procedure was followed for the 197.19 m/z to 75.03 m/z transition, where the cone voltage was optimized at 60 V, while the collision was held at 1 V, and following the collision was optimized to 2 V. The 197.19 m/z to 110.04 m/z cone voltage was tested in intervals, and optimized to 115 V, while the collision was determined to be 5 V. For the 197.19 m/z to 89.05 m/z fragmentation transition, the cone voltage was determined to be optimized at 95 V, and a collision of 4 V. The capillary voltage of 3.5 kV was previously optimized for the other compounds detected in the existing method. The fragmentation 197.19 m/z to 110.04 m/z was selected based on the signal-to-noise ratio in repeated standard injections. A summary of the mass spectrometer parameters used is given in Table S3-1.

TABLE S3-1. Mass spectrometer parameters.

Compound	Parent ion (m/z)	Daughter ion (m/z)	Dwell time (s)	Cone (V)	Collision cell (eV)	Delay time(s)
L-DOPA	197.07	75.03	0.014	60.00	2.00	−1.000
L-DOPA	197.07	89.05	0.014	95.00	4.00	−1.000
L-DOPA	197.07	110.04	0.014	110.00	5.00	−1.000
L-DOPA	197.07	124.05	0.014	15.00	2.00	−1.000

Method validation

The limits of detection and limits of quantification were determined by replicate analysis of standards. The linear range was 0.6–2500 ng on column (Fig. S3-3A). The lower limit of detection was 77 ng on column (Fig. S3-3B).

Data acquisition and processing

Data were acquired by MassLynx version 4.1, processed using TargetLynx version 4.1 (Waters Inc.) and exported to Excel.

LITERATURE CITED

Saremba, B. M., F. J. M. Tymm, K. Baethke, M. R. Rheault, M. Sherif, P. K. Saxena, S. J. Murch, et al. 2017. Plant signals during beetle (*Scolytus multistriatus*) feeding in American elm (*Ulmus americana* Planch.). *Plant Signaling and Behavior* 12: 1–11.

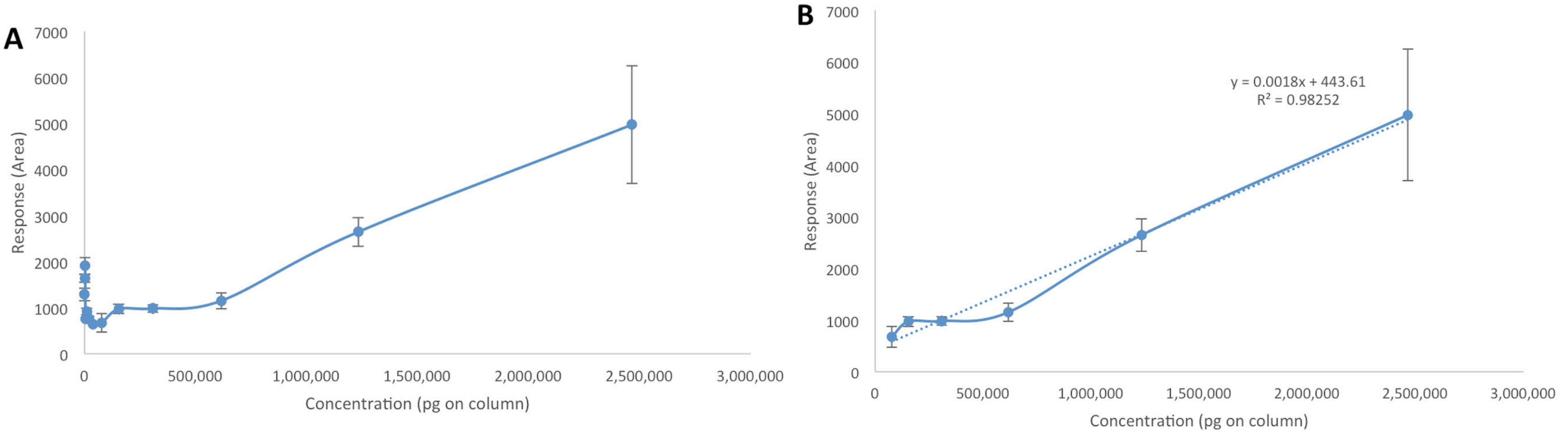

FIGURE S3-3. Determination of linearity, limits of detection, and limits of quantification by repeated injections of standards across a dilution series from 0.6–2500 ng on column, showing the full range of standards (A) and the linear range (B). Note: At the lowest concentration of the full range of standards, the instrument noise is greater than the L-DOPA signal.
