# Supplementary material for: A virus‐induced gene‐silencing system for functional genetics in a betalainic species, Amaranthus tricolor (Amaranthaceae)
Source: Appl Plant Sci. 2019 Feb 7;7(2):e01221. doi: 10.1002/aps3.1221 (PMC6384298; doi:10.1002/aps3.1221)

APPENDIX S4. PCR to confirm the silencing of the target in *Amaranthus tricolor*. (A) Actin was used as an internal control to check the quality of cDNA synthesized from plant tissues. In all panels, a 100-bp DNA ladder was used as the marker (M). Lanes 1–3 indicate the empty vector group replicates, TRV1/TRV2-empty (labeled as EL); lanes 4–6 indicate the uninoculated group replicates (labeled as RL); lanes 7–9 indicate the replicate of the VIGS phenotypic group, TRV1/TRV2-*AtriCYP76AD1* (labeled as VL). Lane 10 is a no template control (labeled as –ve). (B) TRV2-specific primers were used to confirm the presence of viral genome in the EL, RL, and VL groups. Lane 10 is the negative control in the panel. (C) TRV1-specific primers were used to confirm the presence of viral genome in the VIGS plants. Lane 10 shows the amplicons from the positive control, TRV1 plasmid. Lane 11 indicates the no template control. Two TRV-specific targets were selected outside the T-DNA border to test the plasmid contamination on cDNA. (D) NPTII gene primers or kanamycin-resistance gene (represented as KanR in Appendix S1) in lanes 10 and 11 indicate the TRV2 plasmid as the positive control and the no template control, respectively. (E) DNA fragment outside the T-DNA border (TRV2-offBorder) in lanes 10 and 11 indicate plasmid DNA from TRV1 and TRV2 plasmids indicating positive control, and lane 12 shows the no template control.

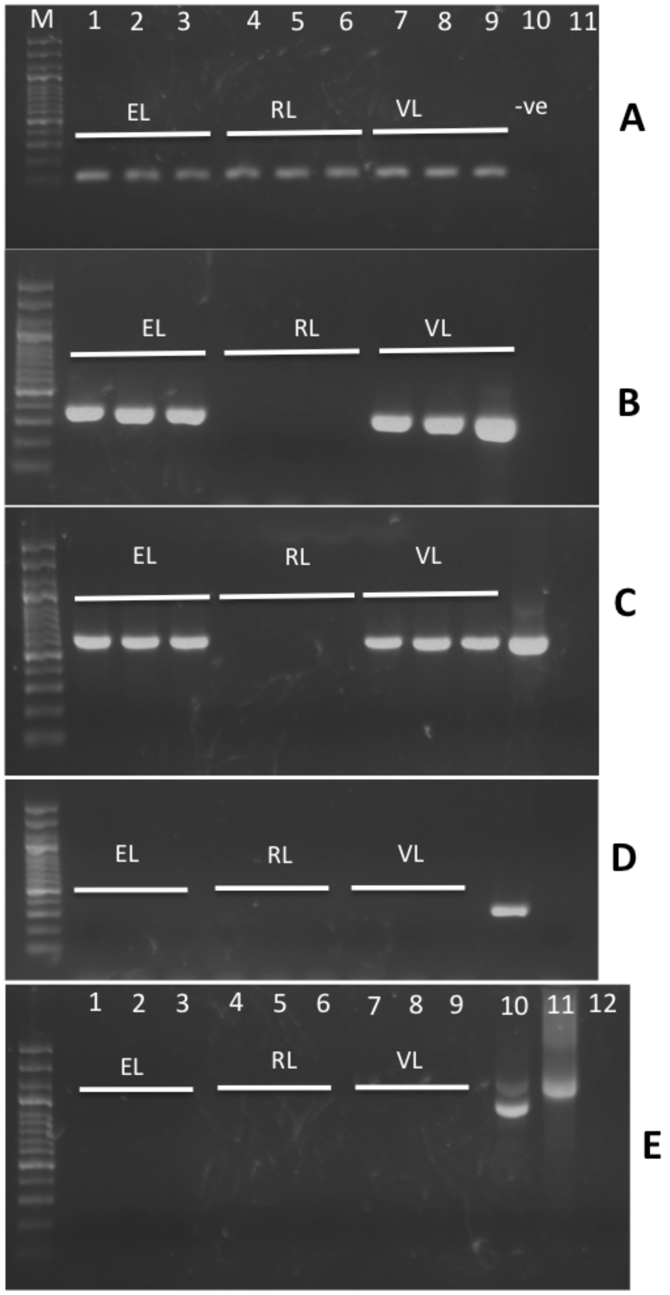

Supplement: Supplementary file 4 — APPENDIX S4. PCR to confirm the silencing of the target in Amaranthus tricolor. (A) Actin was used as an internal control to check the quality of cDNA synthesized from plant tissues. In all panels, a 100‐bp DNA ladder was used as the marker (M). Lanes 1–3 indicate the empty vector group replicates, TRV1/TRV2‐empty (labeled as EL); lanes 4–6 indicate the uninoculated group replicates (labeled as RL); lanes 7–9 indicate the replicate of the VIGS phenotypic group, TRV1/TRV2‐AtriCYP76AD1 (labeled as VL). Lane 10 is a no template control (labeled as –ve). (B) TRV2‐specific primers were used to confirm the presence of viral genome in the EL, RL, and VL groups. Lane 10 is the negative control in the panel. (C) TRV1‐specific primers were used to confirm the presence of viral genome in the VIGS plants. Lane 10 shows the amplicons from the positive control, TRV1 plasmid. Lane 11 indicates the no template control. Two TRV‐specific targets were selected outside the T‐DNA border to test the plasmid contamination on cDNA. (D) NPTII gene primers or kanamycin‐resistance gene (represented as KanR in Appendix S1) in lanes 10 and 11 indicate the TRV2 plasmid as the positive control and the no template control, respectively. (E) DNA fragment outside the T‐DNA border (TRV2‐offBorder) in lanes 10 and 11 indicate plasmid DNA from TRV1 and TRV2 plasmids indicating positive control, and lane 12 shows the no template control. [file APS3-7-e01221-s004.pdf]
