# Supplementary material for: A virus‐induced gene‐silencing system for functional genetics in a betalainic species, Amaranthus tricolor (Amaranthaceae)
Source: Appl Plant Sci. 2019 Feb 7;7(2):e01221. doi: 10.1002/aps3.1221 (PMC6384298; doi:10.1002/aps3.1221)
Supplement: Supplementary file 5 — APPENDIX S5. The DNA sequence of the fragment inserted into pTRV2 to target CYP76AD1 for virus‐induced gene silencing (VIGS). [file APS3-7-e01221-s005.docx]

**APPENDIX S5.** The DNA sequence of the fragment inserted into pTRV2 to target *CYP76AD1* for virus-induced gene silencing (VIGS).

>*AtriCYP76AD1* (contig17897)
ATGGATAATGCAACCCTAGCAATGATACTTACAATATGGCTCATTTCAATTAATTTCATAAAAATGTTTTTTACTCACCAAAACACAAAACTTATTCTTCCTCCGGGTCCAAAACCATTACCAATTATTGGCAACATTCTCGAAGTTGGGAAAAAACCTCATCGTTCATTCGCAAACCTAGCCAAAATCCACGGCCCTTTAATTTCCTTACGTTTAGGAAGTGTTACAACAATTGTCGTATCATCAGCAGAAGTTGCCAAAGAAATGTTCTTAAAAAAAGACCAGCCTCTCTCAAACCGTAATGTTCCCAACTCCGTCACTGCTGGTGATCACCACAAACTAACCATGTCATGGTTACCCGTATCACCCAAATGGAGAAATTTCCGT
