# Supplementary material for: A virus‐induced gene‐silencing system for functional genetics in a betalainic species, Amaranthus tricolor (Amaranthaceae)
Source: Appl Plant Sci. 2019 Feb 7;7(2):e01221. doi: 10.1002/aps3.1221 (PMC6384298; doi:10.1002/aps3.1221)

APPENDIX S6. Multiple sequence alignment of *CYP76AD1*-like gene sequences. The dendrogram is obtained from a maximum likelihood method using nucleotide sequences of *Amaranthus tricolor* transcript sequences (contig74588, contig83066, contig14699, contig17428, contig17897, and contig15583). The dark arrows point at the gene sequences that were used in RT-qPCR for their transcript quantification. * indicates the VIGS target, *AtriCYP76AD1*. Contig74588 and contig14699 are potential homologs of *BvCYP76AD5* and *BvCYP76AD6*, respectively. The betalainic species included in the phylogeny are as follows: KR376385.1 *Froelichia latifolia*; KR376382.1 *Alternanthera caracasana*; KR376378.1 *Alternanthera ficoidea*; KT962274.1 *Beta vulgaris*; KR376381.1 *Beta vulgaris* subsp. *maritima*; HQ656026.1 *Mirabilis jalapa*; HQ656025.1 *Amaranthus cruentus* cv. Kerala; HQ656023.1 *Beta vulgaris* cv. W357B; KC857455.1 *Celosia cristata*; and KM592961.1 *Beta vulgaris*.

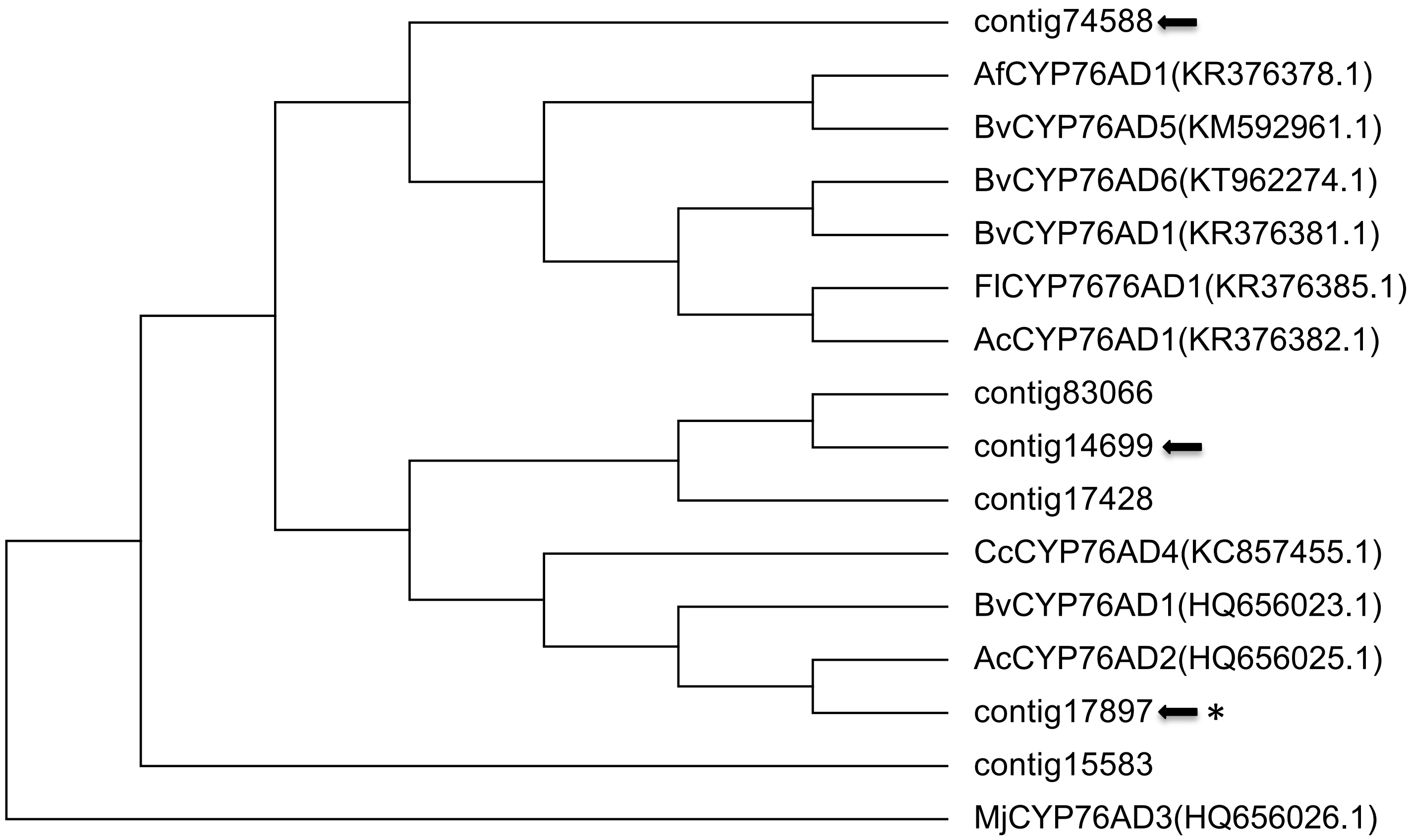

Supplement: Supplementary file 6 — APPENDIX S6. Multiple sequence alignment of CYP76AD1‐like gene sequences. The dendrogram is obtained from a maximum likelihood method using nucleotide sequences of Amaranthus tricolor transcript sequences (contig74588, contig83066, contig14699, contig17428, contig17897, and contig15583). The dark arrows point at the gene sequences that were used in RT‐qPCR for their transcript quantification. * indicates the VIGS target, AtriCYP76AD1. Contig74588 and contig14699 are potential homologs of BvCYP76AD5 and BvCYP76AD6, respectively. The betalainic species included in the phylogeny are as follows: KR376385.1 Froelichia latifolia; KR376382.1 Alternanthera caracasana; KR376378.1 Alternanthera ficoidea; KT962274.1 Beta vulgaris; KR376381.1 Beta vulgaris subsp. maritima; HQ656026.1 Mirabilis jalapa; HQ656025.1 Amaranthus cruentus cv. Kerala; HQ656023.1 Beta vulgaris cv. W357B; KC857455.1 Celosia cristata; and KM592961.1 Beta vulgaris. [file APS3-7-e01221-s006.pdf]
