# Supplementary material for: A virus‐induced gene‐silencing system for functional genetics in a betalainic species, Amaranthus tricolor (Amaranthaceae)
Source: Appl Plant Sci. 2019 Feb 7;7(2):e01221. doi: 10.1002/aps3.1221 (PMC6384298; doi:10.1002/aps3.1221)
Supplement: Supplementary file 7 — APPENDIX S7. Sequence alignment of AtriCYP76AD1 and BvCYP76AD1. [file APS3-7-e01221-s007.rtf]

Adhikary et al.—Applications in Plant Sciences 2019 7(2)—Data Supplement S7DOIAppendix S7. Sequence alignment of AtriCYP76AD1 and BvCYP76AD1. AtriCYP76AD1_17897      MDNATLAMILTIWLISINFIKMFFTHQNTKLILPPGPKPLPIIGNILEVGKKPHRSFANLBvCYP76AD1              MDHATLAMILAIWFISFHFIKLLFSQQTTKL-LPPGPKPLPIIGNILEVGKKPHRSFANL                        **:*******:**:**::***::*::*.*** ****************************AtriCYP76AD1_17897      AKIHGPLISLRLGSVTTIVVSSAEVAKEMFLKKDQPLSNRNVPNSVTAGDHHKLTMSWLPBvCYP76AD1              AKIHGPLISLRLGSVTTIVVSSADVAKEMFLKKDHPLSNRTIPNSVTAGDHHKLTMSWLP                        ***********************:**********:*****.:******************AtriCYP76AD1_17897      VSPKWRNFRKITAVHLLSPLRLDACQSLRQAKVQQLYQYVQECAQKGQSIDIGKAAFTTSBvCYP76AD1              VSPKWRNFRKITAVHLLSPQRLDACQTFRHAKVQQLYEYVQECAQKGQAVDIGKAAFTTS                        ******************* ******::*:*******:**********::**********AtriCYP76AD1_17897      LNLLSKLFFSKELACHKSHESQEFKQLIWNIMEDIGKPNYADYFPILGCIDPLGIRRRLABvCYP76AD1              LNLLSKLFFSVELAHHKSHTSQEFKELIWNIMEDIGKPNYADYFPILGCVDPSGIRRRLA                        ********** *** **** *****:***********************:** *******AtriCYP76AD1_17897      ANFDKLIAVFQTIISERL---ENNKNANATNDVLDVLLQLYKQKELSMGEINHLLVDIFDBvCYP76AD1              CSFDKLIAVFQGIICERLAPDSSTTTTTTTDDVLDVLLQLFKQNELTMGEINHLLVDIFD                        ..********* **.***   .....:.:*:*********:**:**:*************AtriCYP76AD1_17897      AGTDTTSSTFEWVMTELIRNPNMMEKAQQEIQEVLGKDRQIQESDIIKLPYLQALIKETLBvCYP76AD1              AGTDTTSSTFEWVMTELIRNPEMMEKAQEEIKQVLGKDKQIQESDIINLPYLQAIIKETL                        *********************:******:**::*****.********:******:*****AtriCYP76AD1_17897      RLHPPTVFLLPRKADMDVELYGYVVPKDAQILVNLWAIGRDPQAWEKPNAFLPERFLGSDBvCYP76AD1              RLHPPTVFLLPRKADTDVELYGYIVPKDAQILVNLWAIGRDPNAWQNADIFSPERFIGCE                        *************** *******:******************:**::.: * ****:*.:AtriCYP76AD1_17897      VDVKGRDFGLLPFGAGKRICPGMNLAIRMLTLMLATLLQFFDWKLEEGMNPQDLDMDEKFBvCYP76AD1              IDVKGRDFGLLPFGAGRRICPGMNLAIRMLTLMLATLLQFFNWKLEGDISPKDLDMDEKF                        :***************.************************:**** .:.*:********AtriCYP76AD1            GIALQKTKPLEIIPS---BvCYP76AD1              GIALQKTKPLKLIPIPRY                        **********::**  
