# Supplementary material for: A virus‐induced gene‐silencing system for functional genetics in a betalainic species, Amaranthus tricolor (Amaranthaceae)
Source: Appl Plant Sci. 2019 Feb 7;7(2):e01221. doi: 10.1002/aps3.1221 (PMC6384298; doi:10.1002/aps3.1221)

APPENDIX S9. Proposed betalain biosynthetic pathway. Steps 1, 2, and 3 are enzyme mediated; steps 4 and 5 are proposed to be spontaneous. Pathway is redrawn from Hatlestad et al. (2012).

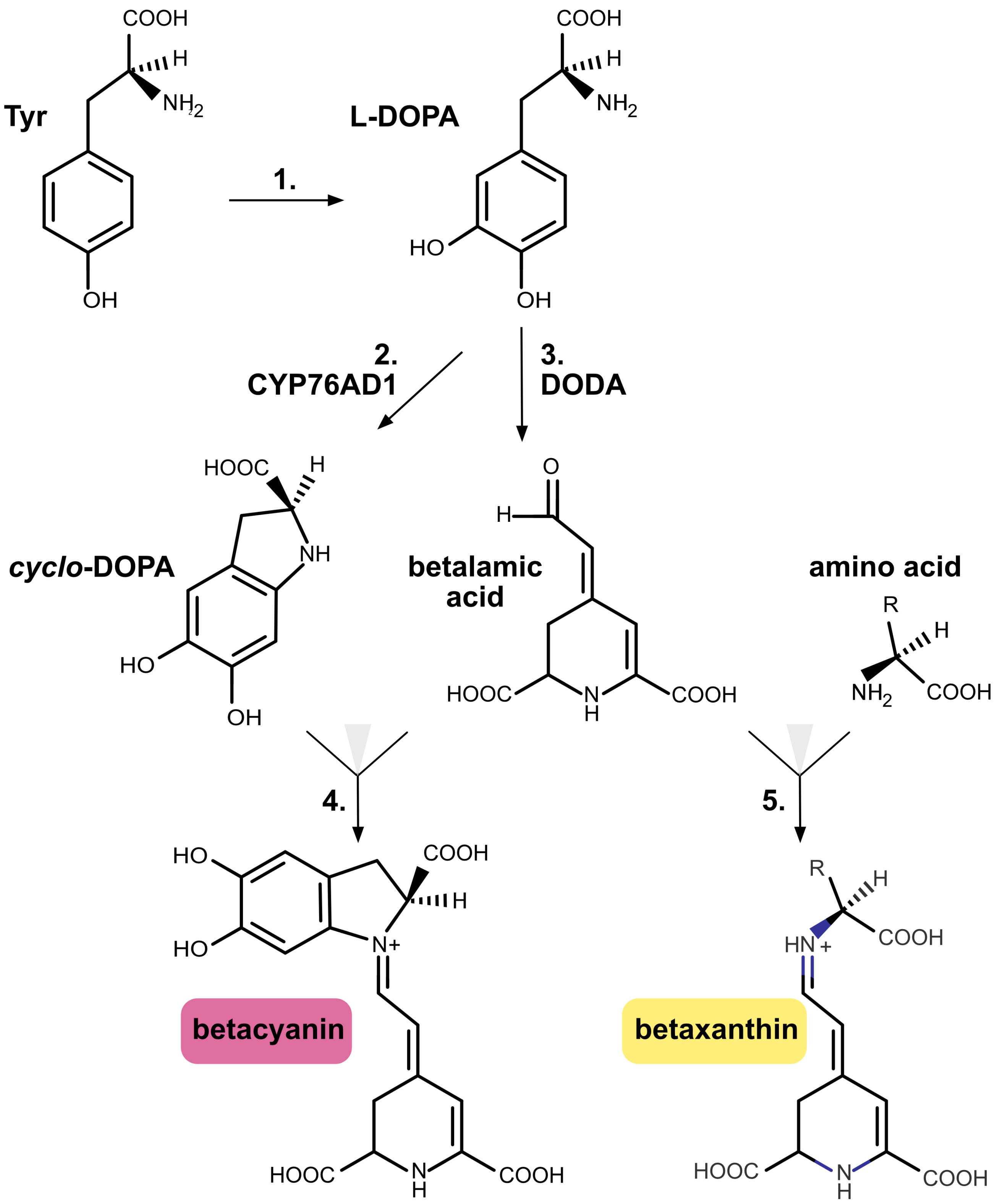

Supplement: Supplementary file 9 — APPENDIX S9. Proposed betalain biosynthetic pathway. Steps 1, 2, and 3 are enzyme mediated; steps 4 and 5 are proposed to be spontaneous. Pathway is redrawn from Hatlestad et al. (2012). [file APS3-7-e01221-s009.pdf]
